# Supplementary material for: T cell receptor repertoires of mice and humans are clustered in similarity networks around conserved public CDR3 sequences
Source: eLife. 2017 Jul 21;6:e22057. doi: 10.7554/eLife.22057 (PMC5553937; doi:10.7554/eLife.22057)
Supplement: Supplementary file 1. — Mouse data: 12 mice from (Madi et al., 2014). Human data: 11 young subjects from (Britanova et al., 2014). DOI: http://dx.doi.org/10.7554/eLife.22057.024 [file elife-22057-supp1.docx]

|  | **Mouse networks**  (1,000 most abundant CDR3β AA sequences) | **Human networks**  (1,000 most abundant CDR3β AA sequences) |
| --- | --- | --- |
| **No. of connected nodes**  **(Random network)** | 647±104  (225±64) | 208±80  (8±4) |
| **No. of edges**  **(Random network)** | 1282±383  (152±52) | 367±201  (4±2) |
| **Correlation: Node degree vs. sequence abundance** | 0.1166 (Spearman) | 0.0008 (Spearman) |
| **Correlation: Node degree vs. sequence sharing** | 0.69 (Spearman) | 0.56 (Spearman) |
| **Correlation: Node betweenes vs. sequence sharing** | 0.58 (Spearman) | 0.43 (Spearman) |

**Table S1.** Statistics of TCR networks for mouse and human repertoires. Mouse data: 12 mice from ([Madi et al. 2014](#_ENREF_12)). Human data: 11 young subjects from ([Britanova et al. 2014](#_ENREF_4)).
