# Supplementary material for: T cell receptor repertoires of mice and humans are clustered in similarity networks around conserved public CDR3 sequences
Source: eLife. 2017 Jul 21;6:e22057. doi: 10.7554/eLife.22057 (PMC5553937; doi:10.7554/eLife.22057)
Supplement: Supplementary file 2. — Connected.nodes and edges refers to network statistics generated from the 1000 most frequent CDR3 sequences in each mouse. DOI: http://dx.doi.org/10.7554/eLife.22057.025 [file elife-22057-supp2.docx]

| **Sample.id** | **connected.nodes** | **edges** | **total.reads** | **unique.NT** | **unique.AA** |
| --- | --- | --- | --- | --- | --- |
| **B6_1** | 967 | 2996 | 721498 | 196253 | 132693 |
| **B6_2** | 948 | 2783 | 281123 | 80249 | 59685 |
| **B6_3** | 909 | 2298 | 143030 | 42919 | 33898 |
| **Quad_1** | 290 | 333 | 855561 | 86568 | 70294 |
| **Quad_2** | 336 | 380 | 504945 | 68993 | 57508 |
| **Quad_3** | 329 | 348 | 899687 | 84125 | 70414 |

**Table S2.** Summary of the data for the quad-KO mice, which are lacking four elements needed for physiological MHC-dependent antigen selection: MHC-I and -II molecules together with CD4 and CD8 co-receptor molecules ([Van Laethem et al. 2007](#_ENREF_34); [Van Laethem et al. 2013](#_ENREF_35)), and matched control WT mice. Connected.nodes and edges refers to network statistics generated from the 1,000 most frequent CDR3 sequences in each mouse.
