# Supplementary material for: T cell receptor repertoires of mice and humans are clustered in similarity networks around conserved public CDR3 sequences
Source: eLife. 2017 Jul 21;6:e22057. doi: 10.7554/eLife.22057 (PMC5553937; doi:10.7554/eLife.22057)
Supplement: Supplementary file 3. — Spleens were harvested on day 7 post immunization and T cells were extracted for TCR analysis. (3) in vitro stimulation: T cells from spleens of immunized mice were harvested on day 7 and were re-stimulated with irradiated splenocytes and the relevant peptide antigen. (4) Five of the OVA-immunized mice received a boost IP injection of 100 μg OVA + CFA on day 14, and spleens were harvested on day 60 for TCR analysis. (5) DN thymocytes. DOI: http://dx.doi.org/10.7554/eLife.22057.026 [file elife-22057-supp3.docx]

| **Sample_ID** | **nt** | **aa** |
| --- | --- | --- |
| **immP277_1** | 16449 | 14914 |
| **immP277_2** | 179380 | 133571 |
| **immP277_3** | 58960 | 50810 |
| **immP277_4** | 84912 | 71310 |
| **immP277_5** | 39360 | 33414 |
| **invitroP277_1** | 38205 | 33385 |
| **invitroP277_2** | 10342 | 9669 |
| **invitroP277_3** | 14198 | 13074 |
| **invitroP277_4** | 44972 | 38018 |
| **invitroP277_5** | 36580 | 32324 |
| **immOVA_1** | 139940 | 106653 |
| **immOVA_2** | 128300 | 99405 |
| **immOVA_3** | 74918 | 61069 |
| **immOVA_4** | 128654 | 102422 |
| **immOVA_5** | 109285 | 87337 |
| **invitroOVA_1** | 25538 | 22702 |
| **invitroOVA_2** | 24408 | 21628 |
| **invitroOVA_3** | 42550 | 36661 |
| **OVA2M_1** | 122362 | 93954 |
| **OVA2M_2** | 77106 | 62947 |
| **OVA2M_3** | 70302 | 58023 |
| **OVA2M_4** | 81666 | 62823 |
| **OVA2M_5** | 60879 | 50825 |
| **dn_1** | 8730 | 8210 |
| **dn_2** | 13413 | 12347 |
| **dn_3** | 19179 | 17994 |

**Table S3.** Summary of TCR-seq data used in this study, from 5 experimental conditions: (1) mice that were immunized with either Chicken Ovalbumin (OVA) or (2) peptide 277 (p277), of HSP60. Spleens were harvested on day 7 post immunization and T cells were extracted for TCR analysis. (3) *in vitro* stimulation: T cells from spleens of immunized mice were harvested on day 7 and were re-stimulated with irradiated splenocytes and the relevant peptide antigen. (4) Five of the OVA-immunized mice received a boost IP injection of 100μg OVA+CFA on day 14, and spleens were harvested on day 60 for TCR analysis. (5) DN thymocytes.
